# Decreased Complex I Activity in Blood lymphocytes Correlates with Idiopathic Pulmonary Fibrosis Severity

**DOI:** 10.1007/s10528-025-11071-w

**Published:** 2025-03-04

**Authors:** Emily Zifa, Sotirios Sinis, Anna-Maria Psarra, Andreas Mouikis, Aglaia Pozantzi, Konstantina Rossi, Foteini Malli, Ilias Dimeas, Paraskevi Kirgou, Konstantinos Gourgoulianis, Ourania S. Kotsiou, Zoe Daniil

**Affiliations:** 1 Department of Biochemistry and Biotechnology, University of Thessaly, Biopolis, 41500 Larissa, Greece; 2 Respiratory Medicine Department, University of Thessaly, Biopolis, 41500 Larissa, Greece; 3 Department of Nursing, University of Thessaly, 41500 Larissa, Greece; 4 Laboratory of Human Pathophysiology, Department of Nursing, University of Thessaly, Gaiopolis, 41110 Larissa, Greece

**Keywords:** Complex I Activity, Fibrosis, Idiopathic Pulmonary Fibrosis (IPF), Mitochondrial Dysfunction, T Cell Immunometabolism

## Abstract

Idiopathic pulmonary fibrosis (IPF) is a progressive interstitial lung disease linked to aging. Mitochondrial dysfunction in circulating T cells, often caused by disruption of mitochondrial DNA (mtDNA), may play a role in age-related conditions like IPF. In our previous study, we found high mtDNA mutational loads in blood lymphocytes from IPF patients, especially in regions critical for mtDNA expression. Since Complex I of the electron transport chain, partly encoded by mtDNA, is essential for energy production, we conducted a preliminary study on its activity. We found significantly reduced Complex I activity (p < 0.001) in lymphocytes from 40 IPF patients compared to 40 controls, which was positively correlated with lung function decline, specifically in functional vital capacity and diffusing capacity for carbon monoxide. These findings indicate that T cell mitochondrial dysfunction is associated with disease progression in IPF. Future work will explore the mechanisms linking T cell mitochondrial disruption with fibrosis, potentially uncovering new therapeutic targets.

**Graphical Abstract:**

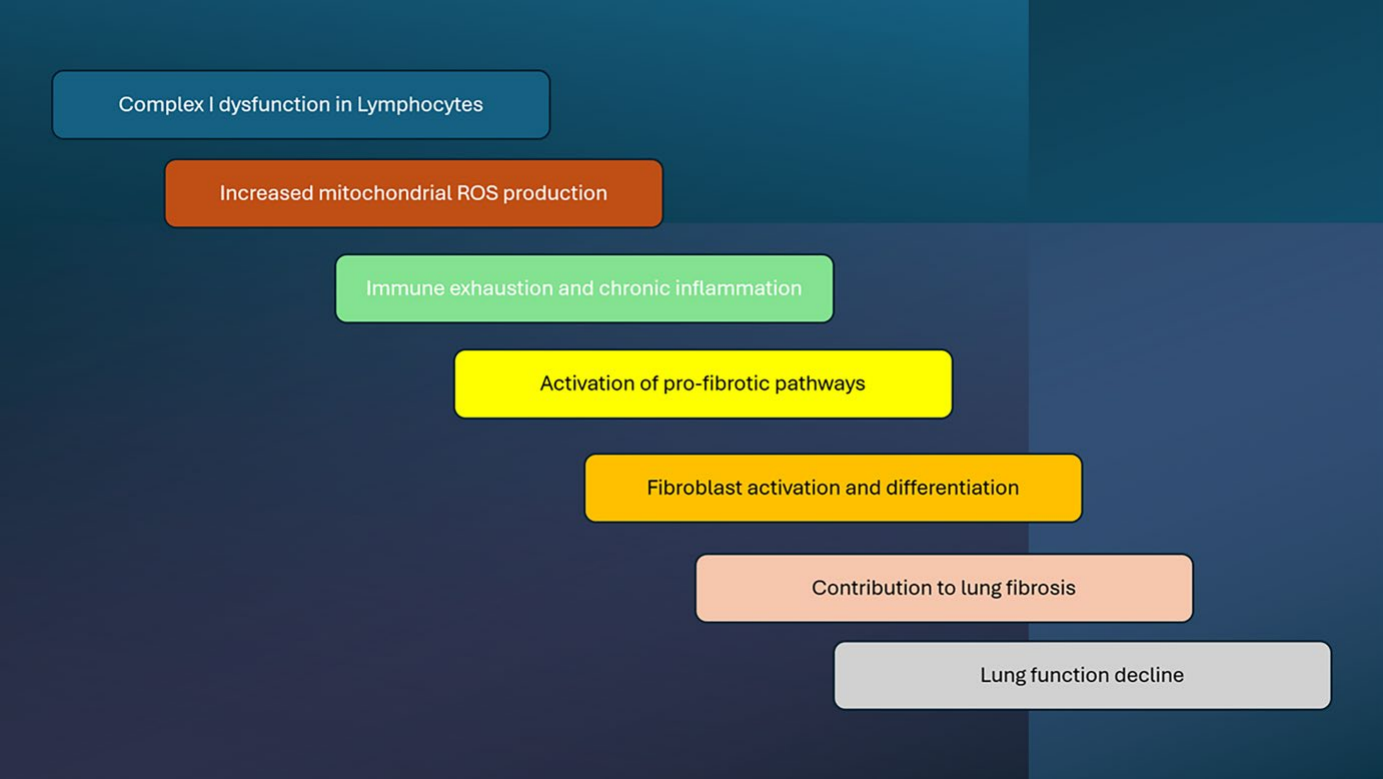

**Supplementary Information:**

The online version contains supplementary material available at 10.1007/s10528-025-11071-w.

## Introduction

Idiopathic pulmonary fibrosis (IPF) is a severe, progressive form of interstitial lung disease that primarily affects elderly individuals. The incidence of IPF in North America and Europe ranges from 2.8 to 9.3 per 100,000 people per year, with a rising trend in both incidence and mortality (Hutchinson et al. 2015). IPF is characterized by persistent epithelial injury and activation, leading to the formation of fibroblastic foci and abnormal wound repair marked by excessive deposition of extracellular matrix (Richeldi et al. 2017). The recent introduction of antifibrotic agents such as pirfenidone and nintedanib has changed the treatment landscape by slowing the decline in pulmonary function (Richeldi et al. 2017). Despite these advances, the prognosis for IPF remains poor, with an average survival of approximately three years, underscoring the urgent need for new therapeutic interventions that target the underlying disease mechanisms (Hutchinson et al. 2015).

Mitochondrial dysfunction has emerged as a significant contributor to IPF pathogenesis. Complex I, a key component of the electron transport chain, plays a crucial role in cellular energy production by facilitating ATP synthesis. Beyond its role in bioenergetics, Complex I also influences apoptosis and reactive oxygen species (ROS) generation, both of which are implicated in fibrotic processes (Sharma et al. 2009). Impaired Complex I function has been linked to age-related diseases, including fibrosis, cancer, and neurodegeneration (Li et al. 2020; Yang et al. 2021; Srivastava 2017 Dec 19). The accumulation of somatic mitochondrial DNA (mtDNA) mutations with aging leads to a loss of mitochondrial function, resulting in decreased energy capacity, increased oxidative damage, and apoptosis. These changes contribute to cellular loss and organ failure (Srivastava 2017 Dec 19). In IPF, mitochondrial dysfunction has been associated with increased ROS production and altered cell survival, contributing to disease progression (Mora et al. 2017; Tsitoura et al. 2019; Bernard et al. 2017; Zhou et al. 2009; Larson-Casey et al. 2016; Desdín-Micó et al. 2020).

Our previous research demonstrated that IPF patients exhibit high mtDNA mutational loads in circulating lymphocytes, particularly in regions critical for mtDNA expression (Daniil et al. 2008, 2018). These mutations could potentially impact mitochondrial function, contributing to disease severity. Therefore, we aimed to assess Complex I activity in lymphocytes from IPF patients compared to healthy controls, to determine if mitochondrial dysfunction is linked to disease severity.

In this study, we also sought to evaluate the correlation between Complex I activity and lung function, specifically forced vital capacity (FVC) and diffusing capacity for carbon monoxide (DLCO). By investigating these relationships, we hope to better understand the role of T cell mitochondrial dysfunction in IPF progression and identify potential targets for therapeutic intervention.

## Methods

### Study Group

Peripheral blood samples were collected from a total of 80 individuals, comprising 40 patients with idiopathic pulmonary fibrosis (IPF) (32 males and 8 females, aged 70.57 ± 10.25 years) and 40 healthy volunteers who served as the control group (28 males and 12 females, aged 62.3 ± 9.9 years). All participants were of Caucasian origin.

A priori power analysis was conducted using *G*Power (version 3.1) to determine the appropriate sample size for this study. Based on previous effect size estimates from mitochondrial dysfunction studies in pulmonary diseases (Desdín-Micó et al. 2020 Jun 19; Bueno et al. 2015 Feb) a sample size of 40 participants per group was required to achieve a statistical power of > 80% at a significance level of α = 0.05. This analysis confirmed that our selected sample size was sufficient to detect significant differences in Complex I activity while minimizing the risk of Type II errors.

The IPF patients were sequentially recruited from the Interstitial Lung Diseases outpatient clinic at the University Hospital of Larissa, Greece, and all met the ATS/ERS/JRS/ALAT criteria for the diagnosis of IPF (Raghu et al. 2018). Patients were enrolled in the study between Jan 2024 and October 2024.

The variables measured included demographic characteristics including age, sex, lung function parameters (FVC, DLCO), medication with antifibrotics and duration of treatment, and Complex I activity levels in lymphocytes. These parameters were analyzed to assess their correlation with Complex I activity.

Healthy volunteers were defined as individuals without IPF. Screening for these volunteers included a thorough review of medical records, blood and urine analyses, electrocardiographic studies, and chest x-rays to ensure their health status.

All participants provided both verbal and written informed consent prior to inclusion in the study, in accordance with the Helsinki Declaration. The study protocol was approved by the Ethics Committee of Larissa University Hospital.

### Isolation of Blood Lymphocytes

Lymphocytes were isolated from blood anticoagulated with 0.18% ethylenediaminetetraacetic acid, using Ficoll-Paque PLUS from Amersham Biosciences, according to manufactures instructions. Briefly, blood (20 ml) was added carefully in a 50 ml tube containing 10 ml Ficoll-Paque. The tube was centrifuged for 20 min at 1800 rpm and 200 C. The lymphocyte-containing layer was collected into a new centrifuge 15 ml tube and diluted with PBS (3 volumes PBS 1X/1 volume lymphocytes collected fluid). After centrifugation for 5 min at 2200 rpm and 20 °C the supernatant was discarded and the cell 100 pellet was resuspended in ice-cold SHE–PIM; 3 volumes SHE-PIM (average 100 μl) / 1 volume 101 cell pellet. SHE-PIM consist of 250 mM sucrose, 10 mM HEPES pH 7.4, and 1 mM 102 ethylenediaminetetraacetic acid supplemented with a protease inhibitor mixture (PIM) of two Complete tablets per 50 ml (Roche). The protein content of blood lymphocytes was determined by the Bio-Rad Protein assay with bovine serum albumin as a standard, and aliquots were rapidly stored at − 80 °C.

### Spectrophotometric Assay

After three cycles of freeze-thawing at − 80 °C and 37 °C (20 min per cycle), permeabilized lymphocytes (70 μg protein/ml final volume) were used to determine respiratory enzyme activity. Complex I activity was measured by monitoring the oxidation of NADH to NAD + at 340 nm, with 380 nm as the reference wavelength, using a spectrophotometer and sub-microcell quartz cuvettes. The assay was conducted in a final volume of 1 ml, using a mixture of 20 mM K_2_HPO_4_ (pH 7.4), 4 mM MgCl_2_, 0.2% bovine serum albumin, 200 μM NADH, 1.7 mM KCN (Complex IV inhibitor), and 3 μM antimycin A (Complex III inhibitor) (Table 1).

**Table 1:** Composition of the spectrophotometric assay mixture. The assay was performed in a final volume of 1 ml

Reagent	Final concentration	Volume added
Κ_2_ΗΡΟ_4_ / pH 7.4	20 mM	200 μl
BSA*	0.2%	100 μl
MgCl_2_	4 mM	8 μl
KCN	1.7 mM	1.7 μl
Antimycin-α	3 μΜ	1 μl
NADH	200 μΜ	35.1 μl
Rotenone	15 μΜ	7.5 μl
CoQ1	100 μΜ	50 μl
Lymphocytes	70 mg prot	100 μl
H_2_O		536.7 μl
		1 ml final

^*^Bovine serum Albumin

For the assay, 850 μl of the mixture and 100 μl of lymphocytes (70 μg protein) were transferred to both a blank cuvette (without rotenone) and a test cuvette (with 5 μl of 15 μM rotenone). To accurately measure Complex I activity and avoid underestimation of rotenone-resistant activity, a separate incubation with rotenone was conducted in parallel. After a 1 min pre-incubation, the reaction was initiated by adding 50 μl of ubiquinone CoQ1 (100 μM final concentration). The decrease in absorbance for 4 min at 340 nm with 380 nm as the reference wavelength was measured (Wit et al. 2007; Birch-Machin and Turnbull 2001).

Complex I activity was determined as the rotenone-sensitive NADH: ubiquinone oxidoreductase activity by subtracting the enzyme activity in the presence of rotenone from that in its absence. The enzymatic activity of Complex I was calculated as nmol/min/mg of protein using the following equation (Spinazzi et al. 2012):

Enzyme activity (nmol/min/mg) = (Δ absorbance_340_/min × Reaction volume 1,000)/ [(extinction coefficient of NADH × volume of sample used in 1 ml)  ×  (sample protein concentration in mg/ml)].

The millimolar extinction coefficient of NADH at 340 nm is 6.22 mmol^−1^ cm^−1^.

### Statistical Analysis

Results are presented as mean ± Standard Deviation (SD) for normally distributed variables, and as median ± SD for non-normally distributed variables. Frequencies of categorical parameters were displayed in pie charts. The Kolmogorov–Smirnov test was used to determine the distribution of continuous variables. Normally distributed continuous variables were compared using Student’s t-test or one-way ANOVA, while non-normally distributed variables were analyzed using the Mann–Whitney U, Wilcoxon, or Kruskal–Wallis tests. The distribution of normal and non-normal variables are presented in supplementary Fig. 1. Categorical data were tested using the Chi-square test, and ordinal data were assessed with Spearman’s rank correlation. Multiple linear regression, adjusting for age, gender, antifibrotic treatment, and duration of treatment, was conducted to assess the relationship between lung function parameters associated with IPF severity and Complex I activity. All data analyses were performed using SPSS (IBM SPSS Statistics, version 25).

## Results

In the IPF group, 80% were male and 20% were female, whereas in the control group, 70% were male and 30% were female. These data are presented in Table 2.

**Table 2:** Epidemiological data of patients and healthy donors and the corresponding average complex I activity

	Controls	IPF	p-value
Number	40	40	
Gender (M/F)	28/12	32/8	0.350
Age ± SD (years)	62.3 ± 9.9	70.57 ± 10.25	0.150
Range (years)	39–80	44–82	
Complex I activity ± SEM (nmol/mg protein/min)	57.18 ± 4.82	10.25 ± 1.91	p < 0.001

*M/F* Male/Female, *N/A* not applicable

### Enzymatic Activity of Complex I in Blood Lymphocytes from Healthy Donors

Using the method described above, we measured Complex I activity in blood lymphocytes from 40 healthy subjects, with an average age of 62.3 ± 9.9 years (range 39–80 years). The average Complex I activity was 57.2 ± 4.8 nmol/mg protein/min, with an interassay variation of 43.44%, consistent with previously published data on human lymphocytes (Bueno et al. 2015 Feb).

While the activity of Complex I typically decreases with age in various tissues, it is noteworthy that no such correlation was observed within the healthy donor group in our study (data not shown) (Srivastava 2017 Dec 19).

### Complex I Activity was Decreased in IPF Lymphocytes Compared to Controls

The activity of ETC Complex I in lymphocytes was significantly lower in the 40 IPF patients (10.25 ± 1.91 nmol/mg protein/min; mean age ± SD: 70.57 ± 10.25 years, range: 44–82 years) compared to the 40 healthy controls (57.18 ± 4.82 nmol/mg protein/min) (Table 2). This decrease was highly significant (p < 0.001) with an effect size of 68%.

### Correlation of Complex I Activity Decrease with Disease Severity

**Fig. 1:**
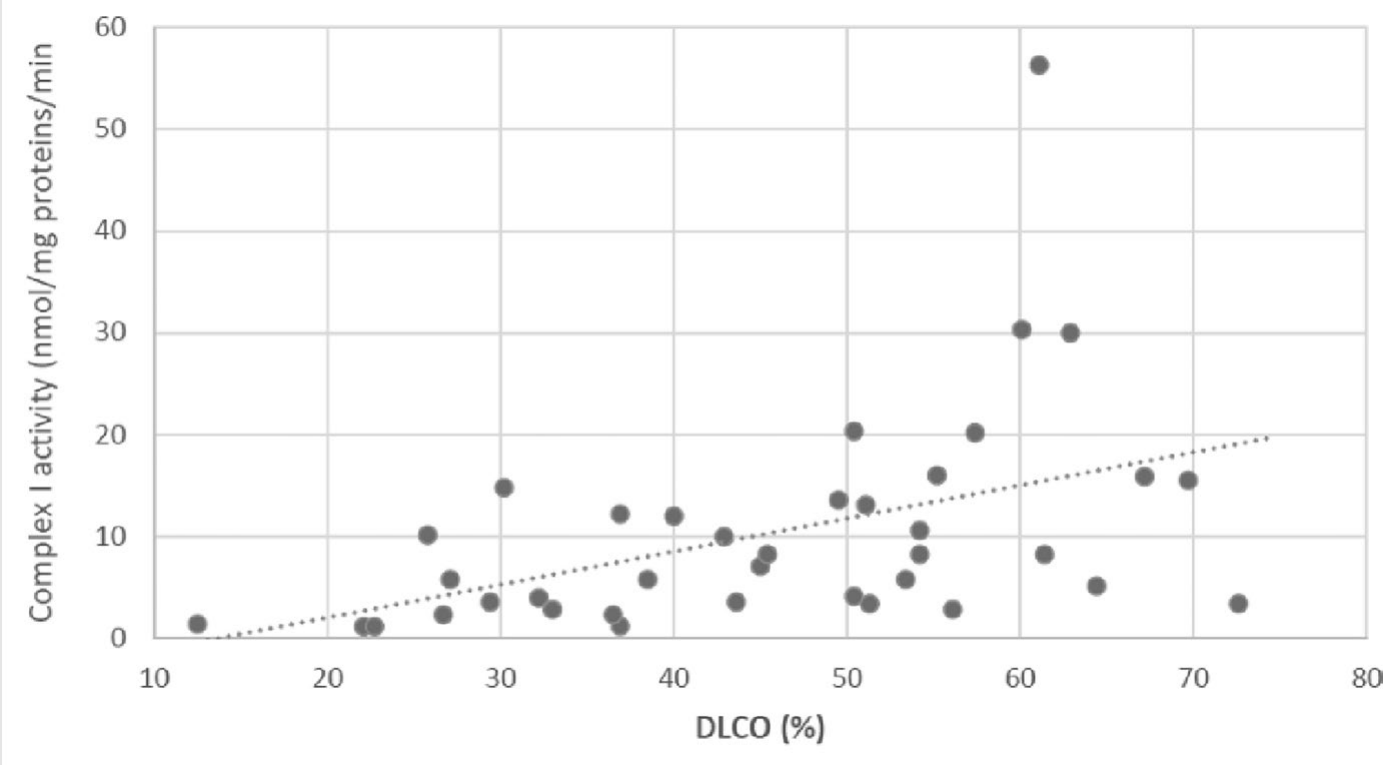
Correlation between complex I activity in circulating lymphocytes of IPF patients and forced vital capacity (% predicted), (p < 0.001, r = 0.868)

**Fig. 2:**
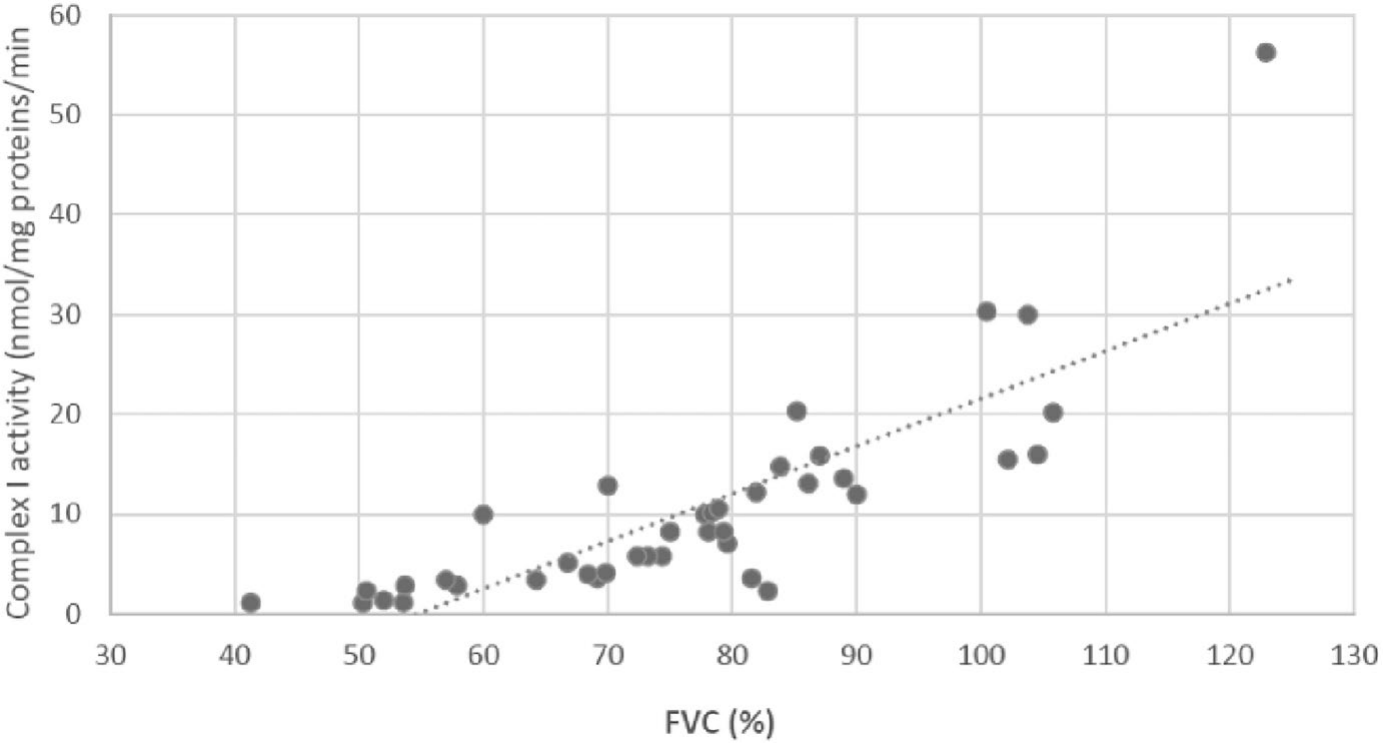
Correlation between complex I activity in circulating lymphocytes of IPF patients and diffusing lung capacity for carbon monoxide (% predicted), (p = 0.001, r = 0.535)

We investigated the correlation of FVC and DLCO with Complex I activity since FVC and DLCO are the most commonly used lung function parameters for denoting functional impairment, progression and response to treatment in IPF (Birch-Machin and Turnbull 2001). We observed a significant positive correlation of complex I activity with FVC (r = 0.868, p < 0.001, Fig. 1) and DLCO (r = 0.535, p = 0.001, Fig. 2). This suggests that reduced mitochondrial function, as indicated by decreased Complex I activity, is closely associated with worsening lung function in IPF patients.

To account for potential confounding effects, additional statistical analyses were conducted using multiple linear regression, adjusting for age, gender, antifibrotic treatment, and duration of treatment to assess the relationship between lung function parameters associated with IPF severity and Complex I activity (Table 3).

**Table 3:** Multiple Linear Regression Analysis Assessing the Relationship Between diffusing capacity for carbon monoxide (DLCO) and Complex I Activity, Adjusted for Age, Gender, and Duration of Antifibrotic Treatment

Model	Unstandardized Coefficients		Standardized Coefficients	t	Sig
	B	Std. Error	Beta		
(Constant)	53.389	18.361		2.908	0.007
Gender	− 5.805	5.493	− 0.170	− 1.057	0.299
Duration of antifibrotic treatment, months	− 0.085	0.128	− 0.107	− 0.663	0.513
Age	− 0.039	0.233	− 0.027	− 0.169	0.867
Complex I activity	0.582	0.217	0.432	2.676	0.012

*Dependent Variable: DLCO. Excluded variables: antifibrotic treatment*

The multiple linear regression analysis assessing the relationship between DLCO and Complex I activity, adjusting for age, gender, and duration of antifibrotic treatment, explained 24.5% of the variance in DLCO (R^2^ = 0.245), with an adjusted R^2^ of 0.144. This suggests that while the model accounts for some variability, additional factors may influence DLCO levels.

Among the predictors, Complex I activity showed a significant positive association with DLCO (β = 0.448, p = 0.009), suggesting that higher Complex I activity is linked to better lung function. Gender was not significantly associated with DLCO (β = − 0.144, p = 0.373), indicating no substantial difference in DLCO between males and females. Duration of antifibrotic treatment had a weak negative association with DLCO (β = − 0.102, p = 0.531), but this was not statistically significant. Age showed a negligible effect on DLCO (β = − 0.025, p = 0.878), indicating that, in this model, age did not strongly influence lung function when accounting for other variables.

Overall, the analysis suggests that Complex I activity is the strongest predictor of DLCO, supporting the hypothesis that mitochondrial function in lymphocytes is linked to lung function impairment in IPF. However, gender, age, and duration of antifibrotic treatment did not significantly impact DLCO in this model.

The multiple linear regression analysis was conducted to assess the relationship between lung function parameters, specifically FVC, and Complex I activity, while adjusting for age, gender, antifibrotic treatment and duration of antifibrotic treatment (Table 4). The model demonstrated a strong predictive capability, explaining 74.8% of the variance in FVC (R^2^ = 0.748), with an adjusted R^2^ of 0.715. The standard error of the estimate was 9.74, suggesting that the model provides a relatively precise estimation of FVC values. Among the predictors, Complex I activity emerged as the most significant factor influencing FVC, with a strong positive association (β = 0.849, p < 0.001). This finding indicates that higher Complex I activity is linked to better lung function, reinforcing the hypothesis that mitochondrial function plays a crucial role in IPF severity. Gender was positively associated with FVC (β = 0.143), suggesting that male participants tended to have higher FVC values than females, although this association did not reach statistical significance (p = 0.131). The duration of antifibrotic treatment showed a minor positive association with FVC (β = 0.057, p = 0.543), but this effect was not statistically significant. Similarly, age had a weak negative effect on FVC (β = − 0.051, p = 0.581), indicating that, in this model, age did not significantly contribute to variations in lung function. Overall, the analysis highlights that Complex I activity is the strongest predictor of FVC, emphasizing the link between mitochondrial function and lung function in IPF patients. The non-significant effects of gender, age, and treatment duration suggest that these factors may have limited influence on FVC when Complex I activity is considered.

**Table 4:** Multiple Linear Regression Analysis Assessing the Relationship Between forced vital capacity (FVC) and Complex I Activity, Adjusted for Age, Gender, and Duration of Antifibrotic Treatment

Model	Unstandardized Coefficients		Standardized Coefficients	t	Sig
	B	Std. Error	Beta		
(Constant)	57.847	13.784		4.197	< 0.001
Gender	6.402	4.124	0.143	1.552	0.131
Duration of antifibrotic treatment, months	0.059	0.096	0.057	0.616	0.543
Age	− 0.098	0.175	− 0.051	-0.558	0.581
Complex I activity	1.502	0.163	0.849	9.201	< 0.001

*Dependent Variable: FVC. Excluded variables: antifibrotic treatment*

## Discussion

This study is the first to report decreased Complex I activity in blood lymphocytes from IPF patients compared to controls, with significant correlations to disease severity. Reduced Complex I activity suggests mitochondrial dysfunction, impacting various lung cell types and contributing to impaired bioenergetics, increased reactive oxygen species (ROS) production, and fibrosis progression (Nathan et al. 2011; Bueno et al. 2020 Jun; Terman et al. 2010; Bueno et al. 2015; Jaeger et al. 2019; Alvarez et al. 2017). Previous studies have shown mitochondrial abnormalities in alveolar epithelial cells (Rangarajan et al. 2017), fibroblasts (Bueno et al. 2020 Jun), and macrophages (Tsitoura et al. 2019), reinforcing the systemic nature of mitochondrial dysfunction in IPF.

Mitochondrial ROS (mtROS) plays a central role in IPF pathogenesis by activating pro-fibrotic pathways, including transforming growth factor-beta (TGF-β), which promotes fibroblast differentiation into myofibroblasts (Liu and Desai 2015 Dec). TGF-β1 downregulates mitochondrial electron transport chain function, particularly at Complex IV, increasing mtROS production. Elevated mtROS further activates latent TGF-β1, creating a self-reinforcing cycle that accelerates fibrogenesis. Additionally, mtROS contributes to epithelial-mesenchymal transition (EMT) and the secretion of pro-fibrotic cytokines, exacerbating tissue remodeling. Myofibroblasts drive extracellular matrix deposition and fibrosis (Hamanaka and Mutlu 2021 Nov; Xie et al. 2015; Schuliga et al. 2018; Gazdhar et al. 2014; Carrasco and Gómez de las Heras MM, Gabandé-Rodríguez E, Desdín-Micó G, Aranda JF, Mittelbrunn M. 2022; Peralta et al. 2024 Dec; Zank et al. 2018 Feb). The accumulation of mtROS in IPF patients' lymphocytes reflects broader immune cell metabolic dysfunction, contributing to systemic inflammation and fibrosis (Hamanaka and Mutlu 2021 Nov; Xie et al. 2015; Schuliga et al. 2018; Gazdhar et al. 2014).

Beyond mtDNA mutations, chronic inflammation and immune exhaustion contribute significantly to mitochondrial dysfunction in IPF. Persistent antigen stimulation leads to T cell exhaustion, characterized by impaired mitochondrial oxidative phosphorylation, reduced glucose uptake, and increased glycolysis reliance (Jiang 2020 Sep 7). These metabolic alterations impair immune surveillance and create a pro-fibrotic environment (Bueno et al. 2020 Jun; Liu and Desai 2015 Dec; Hamanaka and Mutlu 2021 Nov; Xie et al. 2015; Carrasco and Gómez de las Heras MM, Gabandé-Rodríguez E, Desdín-Micó G, Aranda JF, Mittelbrunn M. 2022; Peralta et al. 2024 Dec; Zank et al. 2018 Feb; Jiang 2020 Sep 7).

Dysregulation of mitochondrial quality control further exacerbates mitochondrial dysfunction in IPF. PTEN-induced kinase 1 (PINK1) and Parkin regulate mitophagy, selectively degrading damaged mitochondria. Deficient mitophagy in IPF results in dysfunctional mitochondrial accumulation, increased ROS production, and pro-fibrotic pathway activation (Kobayashi et al. 2016 Jul 15). This impairment may underlie the observed reduction in Complex I activity and contribute to disease progression (Lin et al. 2024 Nov 3).

Type II alveolar epithelial cells (AECII) from IPF lungs exhibit diminished activity of electron transport chain (ETC) complexes I and IV, impairing ATP production and promoting a pro-fibrotic state (Zank et al. 2018 Feb). Similarly, fibroblasts from IPF patients demonstrate reduced ATP production, lower oxygen consumption rates, and elevated mtROS levels (Caporarello et al. 2019 Aug; Yan et al. 2023 Dec 25). In alveolar macrophages, decreased expression of mitochondria-encoded oxidative phosphorylation (OXPHOS) genes and increased mtROS production further contribute to fibrosis (Tsitoura et al. 2019; Rangarajan et al. 2017). These findings confirm that mitochondrial dysfunction is a systemic feature of IPF, affecting multiple lung cell types essential for homeostasis.

Mitochondrial homeostasis disruption is a hallmark of the fibrotic lung and is evident throughout the mitochondrial life cycle. Aging mitochondria exhibit structural abnormalities, including enlargement, cristae loss, and inner membrane destruction, accompanied by deficient ATP synthesis and increased mtROS production (Nathan et al. 2011; Bueno et al. 2020 Jun; Rangarajan et al. 2017). Mitochondrial DNA instability alters ETC component expression, disrupting bioenergetics and contributing to cellular senescence and impaired wound healing in IPF (Terman et al. 2010; Rangarajan et al. 2017). Senescent T cells further exacerbate impaired tissue repair and fibrosis in IPF patients (Bueno et al. 2020 Jun).

A key finding is the marked reduction in Complex I activity within blood lymphocytes of IPF patients compared to healthy controls. This reduction underscores the profound impact of mitochondrial impairment in IPF (Rangarajan et al. 2017). Further analysis reveals a strong positive correlation between Complex I activity and lung function parameters, with FVC and DLCO both positively associated with Complex I activity. This suggests that diminished mitochondrial function is closely linked to the severity of lung impairment in IPF patients (Cala-Garcia et al. 2023 Sep 14). The strong correlation between Complex I activity and lung function highlights mitochondrial dysfunction as a potential biomarker for IPF progression. Reduced Complex I activity in circulating lymphocytes may reflect broader declines in mitochondrial function within lung tissue. A seminal study on age-related multimorbidity in mice demonstrated that T cell metabolic rewiring, driven by mitochondrial DNA instability, plays a significant role in age-related diseases (Amorim et al. 2022 Apr). This parallels our findings of reduced Complex I activity in IPF lymphocytes, suggesting shared pathogenic mechanisms.

This preliminary study has several limitations that may impact the interpretation and generalizability of the findings. First, the small sample size limits the statistical power and may reduce the ability to detect subtle differences or variations in Complex I activity. A larger cohort would be necessary to validate these findings and ensure they are representative of the broader IPF population. Second, the cross-sectional nature of the study precludes any assessment of changes in Complex I activity over time or its direct impact on disease progression. Longitudinal studies are needed to determine whether reduced Complex I activity is a cause or consequence of disease progression. Additionally, the study focuses solely on circulating lymphocytes, which may not fully capture the extent of mitochondrial dysfunction occurring in lung tissue. Further studies investigating mitochondrial function across different cell types involved in IPF, including alveolar epithelial cells and macrophages, would provide a more comprehensive understanding of the disease mechanisms. Despite these limitations, our findings highlight a potential link between mitochondrial dysfunction and IPF severity, warranting further investigation.

Further research is needed to elucidate the mechanistic pathways linking mitochondrial dysfunction to lung function decline in IPF. Future studies should focus on several key areas to build upon our findings. One promising approach is to investigate mitochondrial heterogeneity within different lymphocyte subtypes using advanced techniques such as single-cell RNA sequencing and flow cytometry. This would provide deeper insight into how specific lymphocyte populations contribute to mitochondrial dysfunction in IPF. Additionally, longitudinal studies that track mitochondrial function over time could help clarify whether mitochondrial impairment is a driving factor in disease progression or a consequence of ongoing fibrosis. Experimental approaches that include in vitro and in vivo models could be used to evaluate potential therapeutic interventions aimed at restoring mitochondrial function, such as the use of mitochondrial-targeted antioxidants or agents that enhance mitophagy. Exploring the role of metabolic regulators, such as Sirtuin-1, in modulating T cell function and mitochondrial homeostasis in IPF could also uncover novel targets for intervention (Kim et al. 2022; Deskata et al. 2022). Ultimately, combining these experimental approaches will be crucial for developing effective therapies that address the underlying mitochondrial dysfunction in IPF.

## Conclusion

Our preliminary findings demonstrate that peripheral blood lymphocytes from IPF patients exhibit reduced Complex I activity, correlating with disease severity. These results underscore the need for further studies into T cell metabolic reprogramming and its contribution to pulmonary fibrosis, which could reveal new therapeutic targets for IPF management.

## Supplementary Information

Below is the link to the electronic supplementary material.

Supplementary file1 (TIF 2179 KB)

## Data Availability

Data is provided within the manuscript. The data supporting this research is available from the authors on reasonable request. No datasets were generated or analysed during the current study.
