# Supplementary figures and images for: Decreased Complex I Activity in Blood lymphocytes Correlates with Idiopathic Pulmonary Fibrosis Severity
Source: Biochem Genet. 2025 Mar 4;64(1):1018–31. doi: 10.1007/s10528-025-11071-w (PMC12882963; doi:10.1007/s10528-025-11071-w)

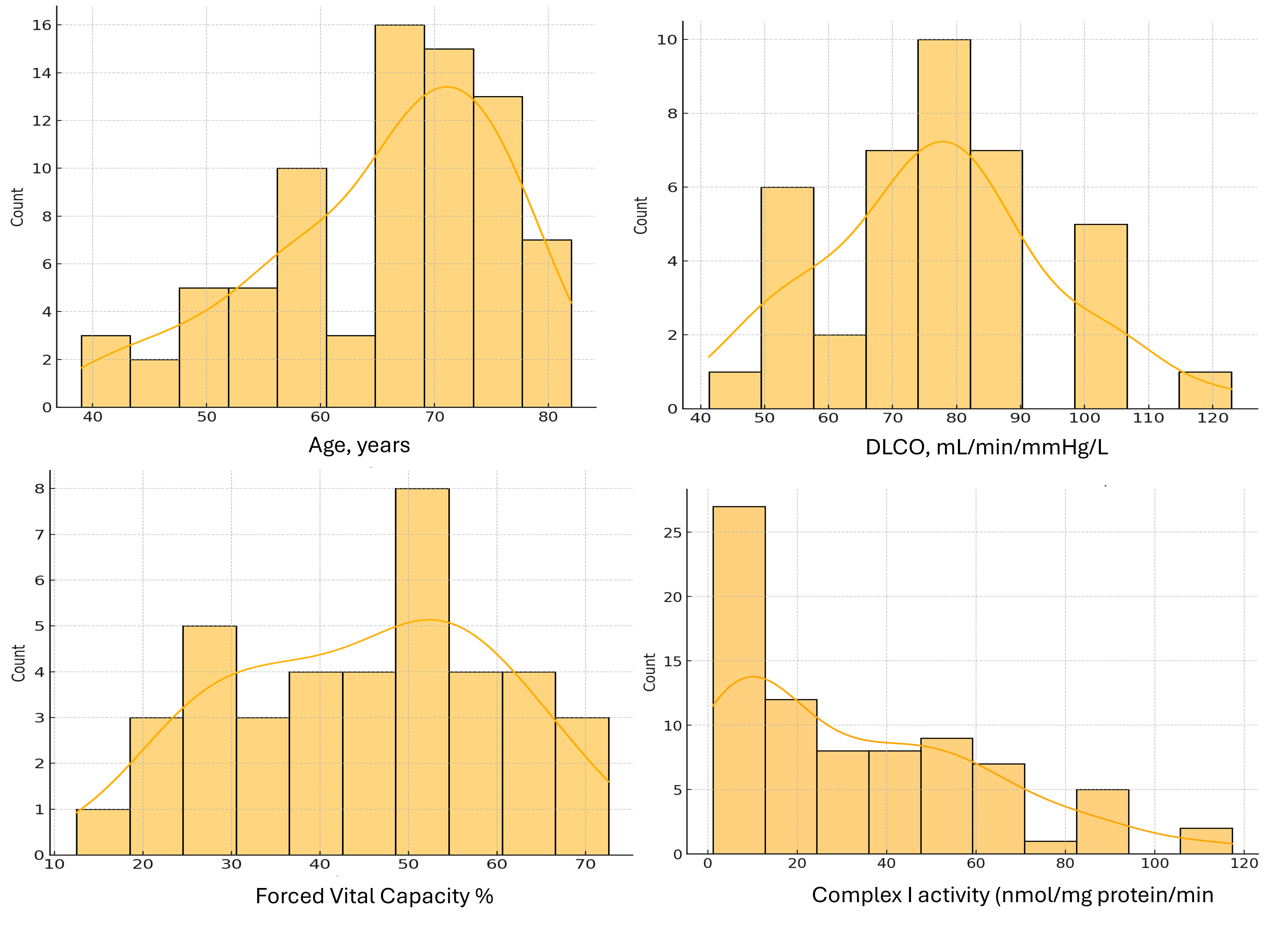

Supplement: Supplementary file 1 — Supplementary file1 (TIF 2179 KB) [file 10528_2025_11071_MOESM1_ESM.tif]
